# Teaching an old dog new tricks: A new tool for protein tyrosine phosphatase substrate discovery

**DOI:** 10.1016/j.jbc.2023.104731

**Published:** 2023-04-18

**Authors:** Anton M. Bennett

**Affiliations:** 1 Yale University School of Medicine, Department of Pharmacology, New Haven, Connecticut, USA; 2 Yale University School of Medicine, Yale Center for Molecular and Systems Metabolism, New Haven, Connecticut, USA

## Abstract

The identification of substrates for protein tyrosine phosphatases (PTPs) is critical for a complete understanding of how these enzymes function. In a recent study in the JBC, Bonham *et al*. developed a modified method combining substrate-trapping mutations with proximity-labeling MS to identify the protein substrates and interactors of PTP1B. This method revealed interaction networks in breast cancer cell models and discovered novel targets of PTP1B that regulate HER2 signaling pathways. This strategy represents a versatile new tool for identifying the functional interactions between PTPs and their substrates.

The balance of protein tyrosine phosphorylation is maintained by the opposing actions of protein tyrosine kinases (PTKs) and protein tyrosine phosphatases (PTPs). The integration of PTKs and PTPs in the regulation of signal transduction pathways is complex, and retention of this balance is essential for cellular homeostasis (1). The significance of PTK–PTP signaling is apparent based on the observations that disruption of this balance leads to human diseases such as cancer and metabolic dysfunction (1, 2). PTPs can represent critical nodal points in signal transduction networks (3). However, the points of PTP integration in these networks are less clear largely due to their poorly defined target substrates.

Much progress has been made in the identification of PTP substrates, yet the quest to identify additional substrates remains challenging (4). The discovery of certain critical molecular determinants of PTP catalysis by Tonks and colleagues (5) has uncovered modes of enzymatic regulation and mechanisms that could be harnessed to facilitate further identification of PTP substrates (6). These studies led to the development of “substrate-trapping” mutations located within the PTP catalytic domain that allowed the enzyme to retain the substrate in a “dead-end” complex (5, 6). However, there are limitations to the substrate-trapping approach. For example, this approach is dependent on the maintenance of low-stoichiometry PTP–substrate complexes during purification, and although the PTP mutants are significantly impaired in activity, there remains sufficient residual activity to disrupt this complex. In addition, the typical approach to introduce the substrate-trapping mutant involved overexpression that generates non-specific PTP–substrate complexes, and the erroneous mislocalization of the PTP mutant confounded interpretation of the physiological relevance of the identified substrates. Taking a new look at these challenges, in a recent issue of the JBC Bonham *et al*. (7) addressed these technical deficiencies with a modernized approach by incorporating a more state-of-the-art technique, proximity-labeling. By combining the “old-trick” of PTP substrate-trapping with a “new-trick” of proximity-labeling, Bonham *et al*. (7) show that PTP1B engages a complex network of substrates and interacting proteins. These proof-of-principle studies demonstrate that this approach should have broad utility across the family of PTPs.

PTP1B has been implicated in the regulation of some substrates that mediates cell proliferation and metabolism (8). Small-molecule PTP1B inhibitors have been shown to block proliferation and induce cell death in HER2-positive and Herceptin-resistant breast cancer (9) but the question of what PTP1B substrates and networks are involved in this inhibitory effect remains unresolved. To find this out, Bonham *et al*. (7) adopted a new approach to identify PTP-substrates in cancer cell models of resistance by combining PTP1B substrate-trapping mutants with proximity labeling (Fig. 1). This approach would not only identify PTP1B substrates but also PTP1B-binding proteins (Fig. 1). MS approaches of the recovered complexes would then yield a “fingerprint” of the PTP1B network of substrates and interactors.

**Figure 1:**
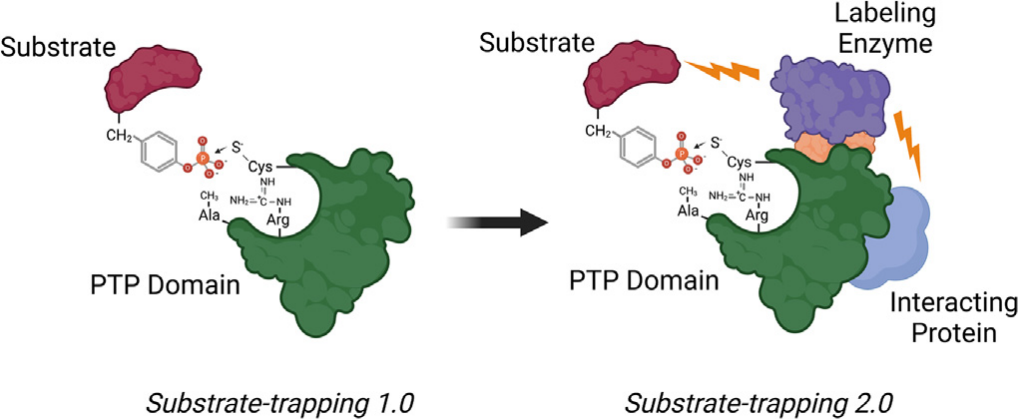
**Evolution of substrate trapping approaches.** The development of substrate-trapping mutant approaches initially utilized mutations within the PTP domain of the acidic aspartic acid residue that is mutated to alanine (Substrate-trapping 1.0). The next generation of PTP substrate-trapping mutants fused a proximity-labeling protein (biotin ligase BirA^R118G^) to generate a chimeric fusion protein (Substrate-trapping 2.0). Proteins bound to the PTP substrate-trapping proximity labeling chimera are biotinylated. The complex can be purified through affinity enrichment using Sepharose-avidin and subjected to MS to identify bound proteins.

To identify potential PTP1B substrates and interacting proteins, the authors fused biotin ligase BirA^R118G^ (BirA^∗^) to the amino-terminus of the substrate-trapping mutant of PTP1B (D181A/C215S). These substrate-trapping PTP1B-BirA^∗^ fusions were expressed in cells and recovered through affinity enrichment with streptavidin Sepharose. The recovered substrate-trapping complexes were subjected to either immunoblotting with anti-phosphotyrosine antibodies or LC-MS. Interestingly, the authors had previously discovered that an allosteric PTP1B inhibitor was effective at preventing the proliferation of acquired and *de novo* Herceptin-resistant breast cancer cells (10). Since these results suggested a role for PTP1B in Herceptin-resistant breast cancer cells, the goal was to use the substrate-trapping PTP1B-BirA^∗^ fusions in these resistant cells to identify potential substrates and interacting partners. An important technical point made by the authors was that the expression levels and proximity labeling kinetics needs to be determined empirically in order to fully optimize biotin-labeling for enrichment and detection by the applied method of detection. It was noted that there was an increased presence of tyrosine-phosphorylated biotin-labeled proteins in substrate-trapping BirA^∗^-PTP1B D181A samples as compared to wild-type BirA^∗^-PTP1B. The authors went one step further and generated PTP1B knockout cells by CRSIPR-Cas9 and performed label-free quantitative proteomics in cells expressing the substrate-trapping BirA^∗^-PTP1B D181A. This approach was validated by the identification of the EGFR as an enriched substrate-trapped interacting protein, along with other known interactors such as RASA2, ARHGAP12, and RNF213. In addition, novel interactors such as CDCP1 were found. CDCP1, not previously known to be either a PTP1B interactor or substrate, is a tyrosine-phosphorylated transmembrane glycoprotein that regulates protein trafficking and has been implicated in breast cancer tumorigenesis, metastasis, and cell migration. The precise role of PTP1B in the regulation of CDCP1 in breast cancer progression remains to be determined; nevertheless, these results provide proof-of-principle that the substrate-trapping BirA^∗^-PTP1B D181A mutant is sufficiently sensitive and robust to identify novel PTP substrates. Finally, the authors showed that the PTP1B substrate-trapping interactome was enriched in proteins involved in cell adhesion, cell matrix, and scaffolding properties, and the vast majority of these proteins were documented to be tyrosine phosphorylated. The regulation of the tyrosine phosphorylation levels of these proteins by PTP1B in acquired and intrinsic Herceptin-resistant cancer cells could suggest applications for PTP1B inhibitors in combination with tyrosine kinase inhibitors for the treatment of HER2-positive breast cancer.

The identification of substrates for PTPs continues to be a daunting task. Substrate-trapping PTP mutants have proven to be a powerful tool, yet with limitations. Combining substrate-trapping PTP mutants with proximity labeling technologies affords new opportunities to identify PTP substrates and interacting partners that might be expressed at low levels in cells. It has long been appreciated that PTPs catalyze the dephosphorylation of their substrates at discrete sub-cellular locations. Following proper optimization, the expression of fused proximity tools with the PTP-substrate trapping mutant at near endogenous expression levels should yield physiologically relevant substrates and interacting proteins. This study demonstrates that this approach could be applicable across the PTP family, thereby providing deeper mechanistic insight into PTP function in signal transduction and human disease.
